# Acute Isolated Corpus Callosum Infarction: A Case Report

**DOI:** 10.7759/cureus.75984

**Published:** 2024-12-18

**Authors:** Nihal Salih, Kholoud Aljaberi, Akhil Narayanan Palat, Sudhir Kumar Palat Chirkkara

**Affiliations:** 1 Neurology, Sheikh Shakhbout Medical City (SSMC), Abu Dhabi, ARE; 2 College of Medicine, Kasturba Medical College of Mangalore, Mangalore, IND; 3 Neurology, Sheikh Shakhbout Medical City (SSMC) and Mayo Clinic, Abu Dhabi, ARE

**Keywords:** corpus callosum, disconnection syndrome, infarction, ischemia, stroke

## Abstract

The corpus callosum is a large subcortical white matter region in the brain that contains fiber connecting both cerebral hemispheres together; it has a rich blood supply; hence, infarction in this region is rare. There are a few reported cases of corpus callosal infarction, and here we present our patient who had a clinical presentation that was not suggestive of corpus callosal infarction. However, an MRI brain confirmed the location, and the patient was managed following the standard stroke treatment, and vascular risk factors were addressed and managed.

## Introduction

Corpus callosal ischemia is uncommon and usually missed clinically. The ability to diagnose this condition has increased with the emergence of newer stroke imaging modalities like diffusion-weighted imaging (DWI) MRI sequence [1]. We could not find any previous case reports from the Middle East and North Africa (MENA) region; hence, we are reporting this case of a 46-year-old male having isolated corpus callosal infarction with a literature review.

## Case presentation

**Figure 1:**
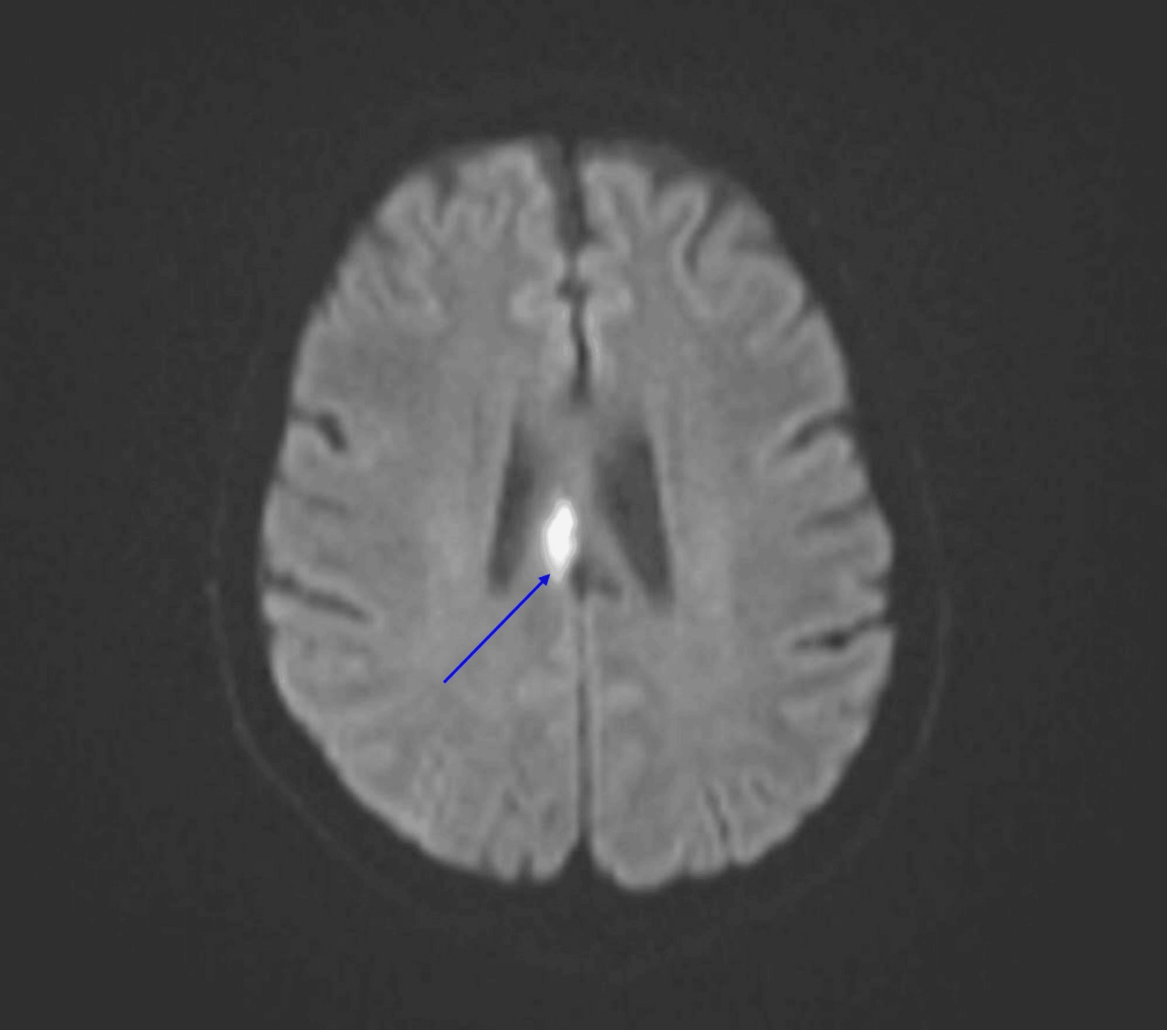
MRI brain, axial view, DWI sequence, showing diffusion restriction in the right body of the corpus callosum. DWI: diffusion-weighted imaging.

**Figure 2:**
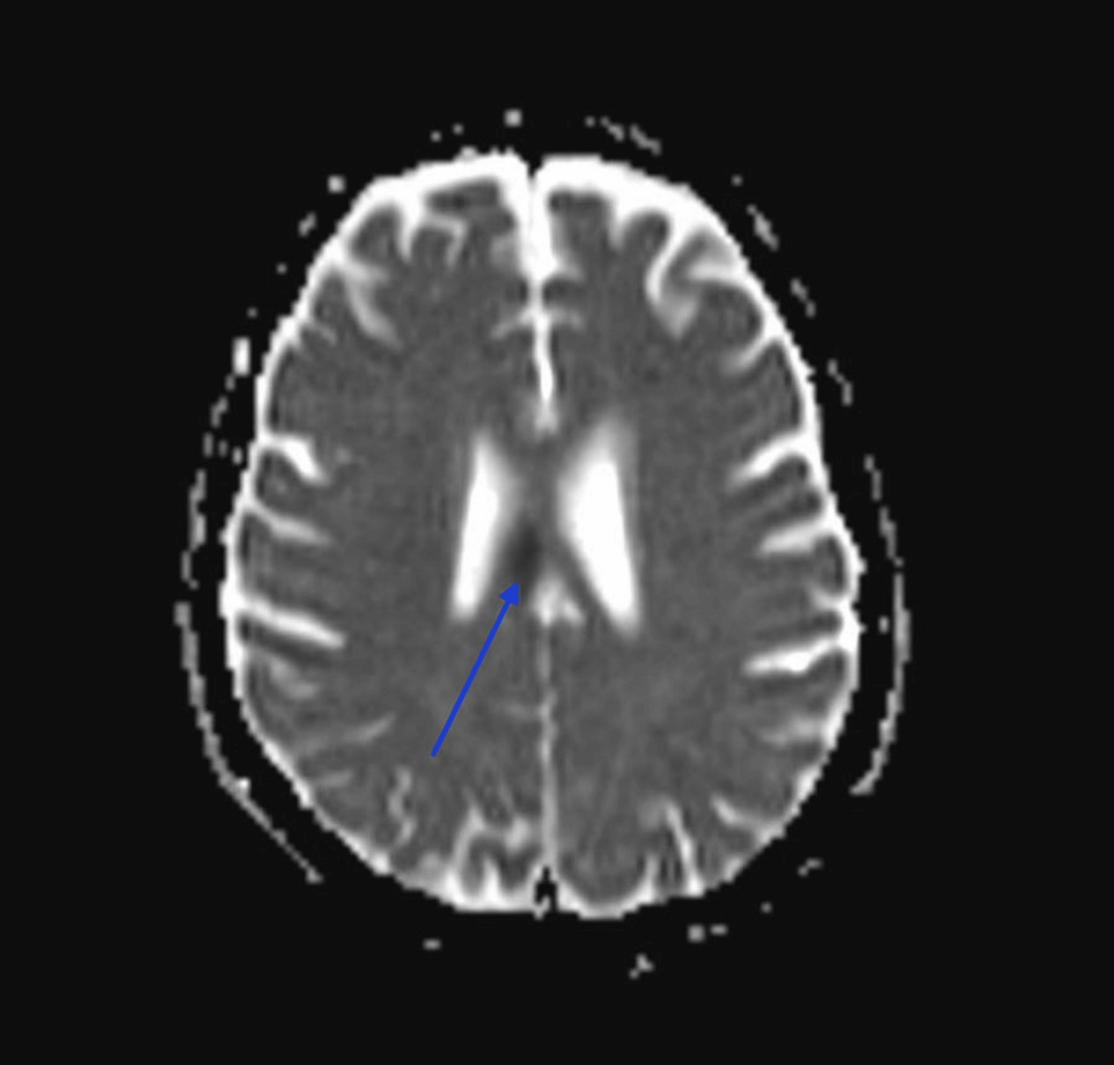
MRI brain, axial view, ADC sequence, showing hypointensity in the right body of the corpus callosum. ADC: apparent diffusion coefficient.

**Figure 3:**
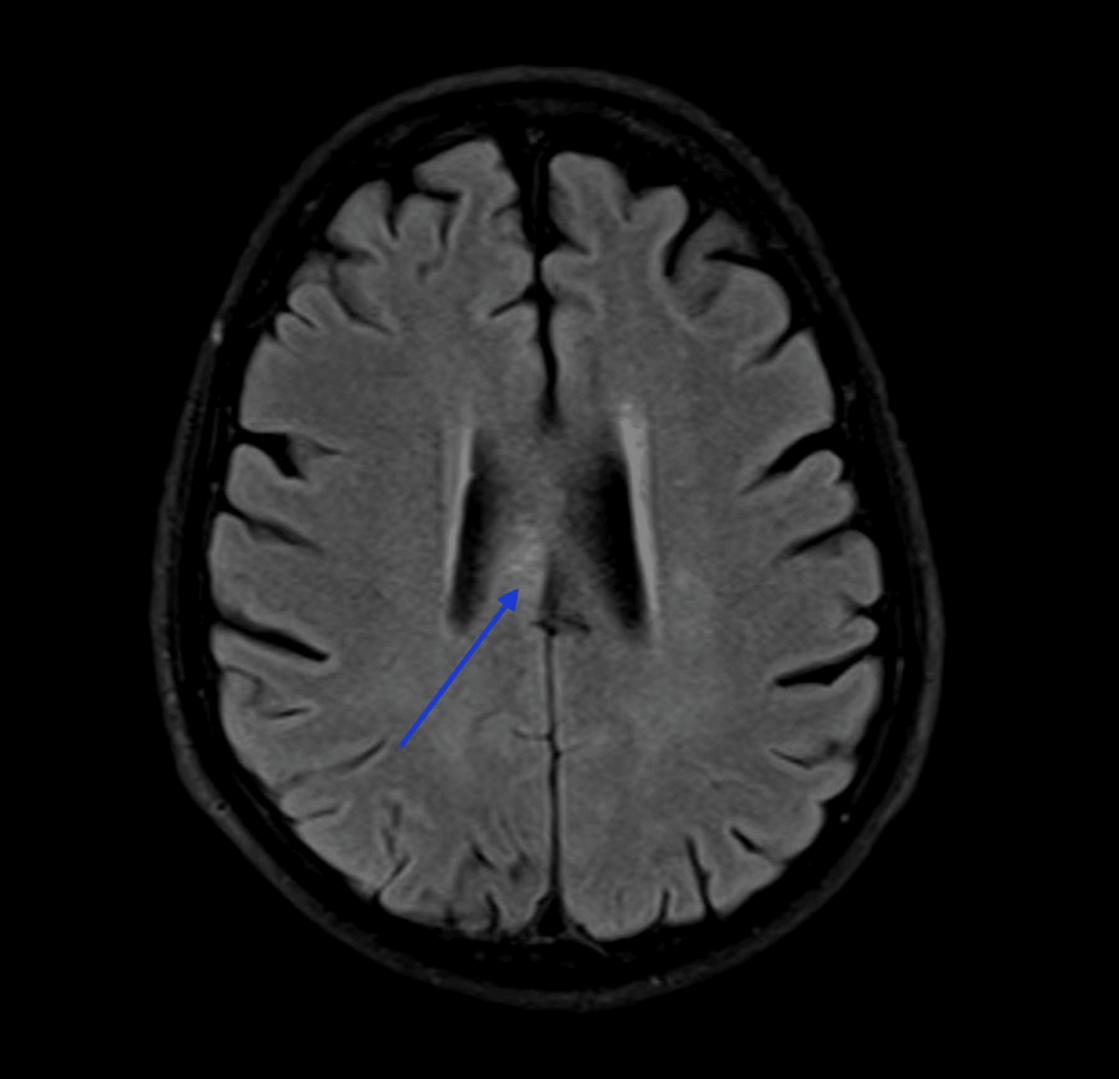
MRI brain, axial view, FLAIR sequence, showing hyper-intensity in the right body of the corpus callosum. FLAIR: fluid-attenuated inversion recovery.

A 46-year-old previously healthy, non-smoker, non-alcohol consumer, male, chef complained of acute onset dizziness for five days in the form of imbalance while walking with a tendency to fall towards his left side. There was no diurnal or postural variation. He also felt a blurring of vision when looking to the left. He had a pulse of 80/minute and 160/100 mm of Hg blood pressure. During the neurological examination, the patient was alert and oriented, with intact cranial nerves 1-12, normal muscle power in upper and lower limbs, and intact sensation for touch, vibration, and proprioception; cerebellar exam revealed dysmetria and overshooting of the left hand while performing the finger nose test, heel-shin test was difficult on the left side, gait assessment showed tendency to sway to left while walking without having wide-based gait, he also had difficulty in performing tandem gait, the patient needed assistance while assessing the gait. The mRS score was three, and the NIHSS was two for the ataxia being present in two limbs. Normal non-contrast CT (NCCT) brain was normal; CT angiography was not done as the patient had the symptoms for more than 24 hours. The MRI brain showed an acute infarct in the body of the right corpus callosum (Figures 1-3). US carotid Doppler showed non-calcified, slightly irregular surface heterogeneous plaques without any significant stenosis in the left carotid bulb extending to the left internal carotid artery (ICA), patent right carotid artery. ECG and echocardiogram were normal. Laboratory values showed HbA1c 6.5% (4.2-6.2), total cholesterol 5.86 mmol/L (3.90-5.20 mmol/L), high-density lipoprotein (HDL) 0.95 (1.10-1.60 mmol/L), low-density lipoprotein (LDL) 3.61 (normal range <2.59 mmol/L), triglyceride (TG) 2.82 mmol/L (0.50-1.70 mmol/L). He was managed with standard vascular risk factor management, and for secondary prevention, aspirin and high-dose atorvastatin were given. At discharge, his mRS score became two, and subsequently, one was on review.

## Discussion

The corpus callosum (Latin for "tough body") is the largest white matter tract containing subcortical commissural fibers connecting both the cerebral hemispheres. It is the largest in terms of size, which is 700 square millimeters for the midsagittal cross-section, and the number of axonal projections, which is 200 million. It integrates and transfers information between cerebral hemispheres in a topographically organized fashion: visual, auditory, and somatosensory information posteriorly and higher cognition anteriorly with brain maturation; it helps to refine motor movements and cognitive functions, including inhibition to prevent alien hand syndrome (AHS) and uncoordinated hand-motor behavior. Anatomically, it is divided into four parts: rostrum, genu, body, and splenium [1,2].

Infarction of corpus callosum is relatively rare, reported between 3% and 8% of all cerebral infarcts [2]. This is attributed to the rich blood supply from anastomosis between anterior and posterior circulations through anterior communicating, anterior cerebral and posterior cerebral arteries, and the perpendicular orientation of the callosal branches to the parent artery. The anterior cerebral artery supplies the rostrum through the subcallosal artery, the body through the pericallosal artery, and the genu through the medial callosal artery. The posterior cerebral artery supplies the splenium through the posterior pericallosal artery. The splenium has been reported to be more commonly involved in embolism, while atherothrombotic infarcts are reported to be more frequent in the genu and body [2,3].

Although the majority of studies showed splenial infarction as the commonest among corpus callosal infarctions (46-55%), some have reported body and genu as well. This could be due to higher infarction incidence in the posterior versus anterior cerebral artery feeding area, racial/ethnic differences, and sample size [2-4]. As per the Trial of Org10172 in Acute Stroke Treatment (TOAST) classification, large-artery atherosclerosis is the most common cause of stroke at 53.3-57.6% in various series [1,4]. The other reported pathologies include cardioembolism and small artery occlusion along rare ones like MoyaMoya disease, intracranial artery dissecting aneurysm, dysplasia of circle of Willis, arteritis, and cryptococcal meningitis. Of late, clusters of splenial infarcts due to COVID-19 have been reported, especially splenial [5,6]. Splenial diffusion restriction can be due to neoplasm, infections, demyelinating disorders, and trauma as well. Nonischemic lesions called cytotoxic lesions of the corpus callosum (CLOCCs) have recently been described [7]. They are usually midline, symmetric, splenial, and often reversible. Causes include brain trauma, metabolic derangements, subarachnoid hemorrhage, infections like viral and certain medication exposures [8].

The risk factors were similar to strokes elsewhere in the brain, with systemic hypertension (72-73.5%), dyslipidemia, diabetes mellitus, and smoking. Coronary heart disease, atrial fibrillation, prior strokes or transient ischemic attack (TIA), excessive alcohol consumption, family history of vascular disease, abnormal hs-CRP values (>5 mg/L), and abnormal homocysteine values (>15 μmol/L) were also reported in lower frequencies [1,4].

The corpus callosal infarction can be unilateral, bilateral, or midline. Clinical manifestations are determined by the extend of a lesion, pure callosal lesions being rare (11-12%) and hence are often nonspecific or overshadowed. Classical symptoms, as described by Giroud and Dumas, are seen with diffuse or bilateral callosal infarct include: (1) callosal disconnection syndrome (CDS) including apraxia, agraphia, tactile anomia of the left hand, and alien hand syndrome (AHS), (2) frontal type gait disorders including a wide base, shuffling gait with short steps and loss of concomitant arm swing as the result of lacunar lesions in the anterior corpus callosum [1-4,9]. AHS, which is a sign of severe isolated corpus callosal infarction, manifests as unconscious movements of the affected hand or incoordination or competitive conflict between both hands. Hemiparesis and sensory dysfunction were the most common associated manifestations of non-callosal lesions. The clinical point is that one is reminded of callosal infarction when the patient has consciousness or cognitive change, apraxia, such as alien hand syndrome with mild paralysis [1,9].

The treatment of corpus callosum infarction is similar to the current ischemic stroke protocol as per American Heart Association (AHA) 2019 guidelines [10], focusing on vascular risk factor modification. The prognosis depends on the patient’s overall vascular risk factor profile, as well as infarct volume and extend of location. A six-month prognosis is generally better when compared to other isolated supratentorial subcortical infarctions due to the abundant blood supply and multiple collateral circulation. Also, these arcuate fibers mainly undergo demyelination rather than neuronal degeneration or necrosis after infarction, and hence, as the ischemia improves, it promotes rapid remyelination [1,4]. Multiple cerebrovascular stenosis, diffuse or large infarction, and diabetes are associated with poor prognosis [1].

## Conclusions

Infarction in the corpus callosum is rare due to the rich blood supply to the region, and isolated infarction without the involvement of other parts of the brain is even rare. The classical and well-recognized presentation is disconnection syndrome. We presented a case of a 45-year-old male with atypical presentation of corpus callosal infarction, as he presented with left-sided ataxia, which is more suggestive of a posterior circulation stroke rather than a corpus callosal infarction. MRI DWI sequence helped to pick the lesion. The patient was given aspirin and atorvastatin, and other vascular risk factors like diabetes were also addressed and managed. 
